# Adding Vitamin E-TPGS to the Formulation of Genexol-PM: Specially Mixed Micelles Improve Drug-Loading Ability and Cytotoxicity against Multidrug-Resistant Tumors Significantly

**DOI:** 10.1371/journal.pone.0120129

**Published:** 2015-04-01

**Authors:** Zhuoyang Fan, Cheng Chen, Xiaoying Pang, Zhou Yu, Yang Qi, Xinyi Chen, Huihui Liang, Xiaoling Fang, Xianyi Sha

**Affiliations:** Key Laboratory of Smart Drug Delivery (Fudan University), Ministry of Education & PLA, Department of Pharmaceutics, School of Pharmacy, Fudan University, Lane 826, Zhangheng Road, Shanghai 201203, China; University of South Florida, UNITED STATES

## Abstract

Genexol-PM, produced by Samyang Company (Korea) is an excellent preparation of paclitaxel (PTX) for clinical cancer treatment. However, it cannot resolve the issue of multidrug resistance (MDR)—a significant problem in the administration of PTX to cancer patients. To increase the efficacy of Genexol-PM against MDR tumors, a mixed micelle capable of serving as a vehicle for PTX was developed, and two substances were chosen as carrier materials: 1) Polyethylene glycol–polylactic acid (PEG-PLA), the original vehicle of Genexol-PM. 2) Vitamin E-TPGS, an inhibitor of P-glycoprotein (P-gp). P-gp has been proven to be the main cause of MDR. *In vitro* evaluation indicated that the mixed micelle was an ideal PTX delivery system for the treatment of MDR tumors; the mixed micelle also showed a significantly better drug-loading coefficient than Genexol-PM.

## Introduction

Tumors are a great threat to human health, and paclitaxel (PTX) is one of the most important anticancer drugs in clinical use today. PTX inhibits microtubule depolymerization of free tubulins; and it has shown its potency against a variety of tumors [1,2]. However, because of its poor aqueous solubility, its clinical use is limited. Its widely used commercial preparation, Taxol, contains polyoxyethylene castor oil (Cremophor EL) and Tween 80 as surfactants to increase the solubility of PTX. However, the high concentration of Cremophor EL may lead to serious side effects, such as hematological toxicity and hypersensitivity [3].

Various drug delivery systems have been developed to replace the vehicle of Taxol to overcome the aforementioned limits. These new delivery systems are mostly aqueous media, such as polymeric micelles [4,5]. Genexol-PM is such a polymeric micelle. It was developed in Korea by Samyang Company. It is mainly used to treat breast and lung cancers, and it is considered as a strong potential alternative to Taxol [6]. It uses PEG-PLA, an amphiphilic block copolymer as carrier to deliver PTX. PTX can be incorporated into the hydrophobic core of the micelle, which is spontaneously formed by PEG-PLA. The hydrophilic shell can help to prevent uptake by the reticuloendothelial system (RES) and increase circulation time [7]. Some research has shown that Genexol-PM can increase the maximum tolerated dose and the median lethal dose of PTX for mice [8,9]. However, Genexol-PM has not solved the problem of multidrug resistance (MDR), a significant obstacle in the chemotherapy of cancer [10]. MDR is caused by the overexpression of proteins of the ATP-binding cassette transporter family in tumor cells, including P-glycoprotein (P-gp) and other multidrug-resistance-associated proteins (MRPs), which mediate the drug efflux [11,12]. PTX is a known substrate of P-gp, and P-gp is mainly responsible for the MDR problem in PTX delivery [13,14]. MDR may be overcome by limiting the activity of P-gp; this may further enhance the antitumor efficacy of Genexol-PM.

To retain the advantages of Genexol-PM, its PEG-PLA vehicle had to be preserved and another amphiphilic block copolymer capable of overcoming the MDR and had properties similar to those of the PEG-PLA had to be found. Previous studies have shown that some pharmaceutical excipients can reverse the MDR property of tumors [15,16], including some small molecule surfactants, such as Tween80 [17], and some amphiphilic block copolymers, such as Pluronic [18]. Here, PEG-PLA and Vitamin E-TPGS (D-α-tocopherol polyethylene glycol 1000 succinate) were used to develop a mixed micelle as a new PTX delivery system. The critical challenge, and the necessary precondition to form a mixed micelle, is the selection of a polymer with a chain length and a block ratio similar to those of the PEG-PLA [19,20]. An ideal mixed micelle can even compensate for the low drug-loading coefficient (DL%), dilution intolerance, and other shortcomings of ordinary micelles. It was here discovered that Vitamin E-TPGS is the optimal choice: It is not only an inhibitor of P-gp [21], but also has a short chain length like the PEG-PLA used in Genexol-PM. Vitamin E-TPGS is obtained by esterification of D-α-Vitamin E-TPGS and PEG1000. It has a water-soluble amphiphilic molecular structure, a large surface area, and a low critical micellar concentration (CMC). Vitamin E-TPGS is biocompatible and is recognized as a pharmaceutical excipient by the Food and Drug Administration (FDA) [22]. A previous study showed that Vitamin E-TPGS has the strongest ability to overcome MDR of any of the various widely used surfactants, including Cremophor EL, Tween 80, and Pluronic P85 [23,24]. Vitamin E-TPGS inhibits drug efflux through allosteric regulation of the P-gp ATP enzyme, thus blocking the energy source of the P-gp [25].

The purpose of this study is to develop a mixed micelle composed of PEG-PLA and Vitamin E-TPGS, incorporating PTX in the core. It may improve the anticancer efficacy by overcoming the MDR of tumor cells. The PTX-loaded mixed micelle was prepared using a thin-film hydration method and its formulation was optimized. Tumor cells were used to verify the improvement *in vitro*.

## Materials and Methods

### 2.1 Materials and cells

PTX was purchased from Xi’an Sanjiang Bio-Engineering Co., Ltd. (Xi’an, China). Methoxy poly (ethylene glycol) (mPEG-OH, Mn is 2.0 kDa) was obtained from JenKem technology Co., Ltd. (Beijing, China). Vitamin E-TPGS was obtained from Peboc (Anglesey, U.K.). Coumarin 6, Vinblastine, Tween 80, and 3-(4,5-dimethyl-thiazol-2-yl)-2,5-diphenyl-tetrazolium bromide (MTT) were purchased from Sigma–Aldrich, Inc. (St. Louis, MO, U.S.). Hoechst 33342 and Lyso Tracker Red were purchased from Beyotime Biotechnology Co., Ltd. (Nantong, China). Cellulose ester membranes (dialysis bag) with a molecular weight cut-off value (MWCO) of 3500 Da (Greenbird Inc., Shanghai, China) were used in the dialysis experiments. Acetonitrile and methanol were supplied by Burdick and Jackson Corp. (Muskegon, MI, U.S.). Dulbecco’s Modified Eagle’s Medium (DMEM), RPMI 1640, fetal bovine serum (FBS), Penicillin-streptomycin, and a 0.25% (w/v) trypsin–0.03% (w/v) EDTA solution were purchased from Gibco BRL (Gaithersberg, MD, U.S.). Deionized water was prepared with a Millipore water purification system (Millipore Corporation, MA, U.S.). Acetonitrile was High Performance Liquid Chromatography (HPLC) grade, and all other chemicals were reagent grade and used without further purification.

The human lung adenocarcinoma cell line A549, human oral epithelial cancer cell line KB, and its vinblastine-resistant subclone KBv were obtained from the Cell Resource Center of the China Science Academy, Shanghai, China. All cells were cultured at 37°C with 5% CO_2_ under fully humidified conditions, supplemented with 10% FBS, 100 U/ml of penicillin, and 100 μg/ml of streptomycin. A549 was cultured in DMEM; KB and KBv were cultured in RPMI 1640. In addition, the KBv culture was supplemented with vinblastine in the medium, the final concentration of which was 200 ng/ml.

### 2.2 Amphiphilic block ratio of PEG-PLA

The amphiphilic block ratio of the PEG-PLA is critical to the micelle formation even for mixed micelles [26]. Three kinds of PEG_2000_-PLA, each with a different molecular weight, were synthesized (PEG/PLA = 70/30, 54/46, 38/62) (Table 1). The encapsulation efficiency (EE%) and DL% of the PTX were used to evaluate the effect on PEG-PLA micelles at different PTX feeding ratios (PTX/PEG_2000_-PLA, w/w). These were calculated as follows.

$$\begin{array}{c} \text{EE\%}=\frac{\text{amount of PTX in micelles}}{\text{amount of the feeding PTX}}\\ \text{DL\%}=\frac{\text{amount of PTX in micelles}}{\text{amount of the feeding polymer and PTX}} \end{array}$$

**Table 1:** PEG_2000_-PLA with different block ratios.

Groups	PEG_2000_–PLA (M.W.)	PEG(M.W.)	PLA(M.W.)	PEG/PLA
**1**	2847	2000	847	70/30
**2**	3678	2000	1678	54/46
**3**	5230	2000	3230	38/62

### 2.3 Preparation of PTX-loaded mixed micelle

The PTX-loaded mixed micelles were prepared by a thin-film hydration technique [5]. Briefly, 25 mg of the mixture composed of PEG-PLA and Vitamin E-TPGS (VE-TPGS) in different proportions was co-dissolved in 4 ml of acetonitrile in a round-bottom flask, and the PTX was added at different ratios (PTX/materials, w/w) to the carrier materials. The solvent was subsequently rotary evaporated to obtain a thin film, which was then kept in a vacuum overnight at room temperature to remove the residual solvent. After adding 10 ml of deionized water and stirring at 500 rpm for 10 min, a clear micelle solution was formed. Then the solution was filtered by a 20 μm filter membrane to separate unincorporated crystalline PTX, and then the lyophilization was performed. The concentration of PTX was determined with HPLC assay after diluting the micelles with acetonitrile.

### 2.4 Formulation

After a series of preliminary experiments to assess the control factors and their corresponding levels, the VE-TPGS mass fraction and the amount of PTX fed in were selected as two variables at five levels and used to optimize the formulation process; particle size, EE%, DL%, and the relative amount of PTX precipitated from micelles after 24 h of constant temperature oscillation at 37°C (PTX Pre%) were taken as four responses. PTX Pre% value was calculated using the following equation:

$$\text{PTX Pre\%}=\frac{\text{original feeding of PTX(mg)}\;-\;\text{PTX remained in the supernatan t(mg)}}{\text{original feeding of PTX(mg)}}$$

The effects of the variables were studied with a central composite design (CCD) [27,28], and thirteen experiment runs were formed according to Design Expert 8.0 software (Stat-Ease, Inc., Minneapolis, MN, U.S.). All of the following statistical analyses of the results of the experiments, including numerical optimization, were conducted by the software.

### 2.5 Characterization of PTX-loaded mixed micelle

#### 2.5.1 Particle size, zeta potential, and morphology of the mixed micelle

The mean particle size and zeta potential of the PTX-loaded PEG-PLA micelle and the PTX-loaded mixed micelle were measured using dynamic light scattering (DLS) analysis with a Zeta Potential and Particle Sizer (Malvern, Nano-ZS, Malvern Instruments Ltd.; Worcestershire, U.K.). The morphological examination of micelles was performed under a transmission electron microscope (TEM, Philips CM120, Philips, the Netherlands) after negative staining with a phosphotungstic acid solution (2.0%, w/v).

#### 2.5.2 Critical micelle concentration determination

The critical micelle concentrations (CMC) of PEG-PLA, VE-TPGS, and their mixture (VE-TPGS/PEG-PLA = 13/87, w/w) were determined using iodine UV spectroscopy as described previously [29]. A set of polymer solutions at various concentrations (1 ml, 0.0001% to 0.1%, w/v) was prepared with 5 μl of KI/I_2_ standard solution added. The UV absorbance values of the polymer solutions were measured at 366 nm with a UV-VIS spectrometer (Shimadzu UV-2401; Shimadzu, Tokyo, Japan). The intensity of absorption was plotted against the logarithm of the polymer mass concentration.

#### 2.5.3 Differential scanning calorimetry

To investigate the incorporated form of the PTX and the polymers, approximately 3 mg of PTX, blank mixed micelle, and PTX-loaded micelle were sealed separately in standard aluminum pans with lids, and subjected to differential scanning calorimetry (DSC 204; Netzsch, Germany). The temperature ramp speed was set at 10°C/min (from 100 to 300°C).

#### 2.5.4 Release profiles ***in vitro***

In order to compare the release behaviors of PEG-PLA micelles and mixed micelles *in vitro*, phosphate buttered saline (PBS, pH7.4) with 0.5% Tween 80 was chosen as the medium to create a pseudo-sink environment [30]. One microliter of PTX-loaded micelle was introduced into a dialysis bag (MWCO = 3500 Da; Greenbird Inc.; Shanghai, China); the sealed end of the bag was completely submerged in 39 ml of the medium at 37°C and stirred at 100 rpm for 24 h. The release behavior of the PTX stock solution was monitored as control. At appropriate intervals, 0.5 ml aliquots were withdrawn and replaced with equal volumes of fresh medium.

The concentration of the PTX in the samples was determined with the HPLC. The machine was a Shimadzu HPLC system equipped with an SIL-10AF auto sampler, an LC-15C pump, a reversed-phase column (Gemini 5 μm C_18_, 200 mm × 4.6 mm, Phenomenex, CA, U.S.), and an SPD-10AVP UV detector (Shimadzu, Kyoto, Japan); and operated at 230 nm. The mobile phase was a mixture of acetonitrile/ water (60:40, v/v). The flow rate was maintained at 1 ml/min. The PTX concentrations were determined by comparing the peak areas with the standard curve.

### 2.6 Cellular association of coumarin-6-labeled mixed micelle

Coumarin-6 was chosen as a fluorescence probe loaded with micelles for assessment of cell uptake. The method of preparation described in *2*.*3* was used. A549 cells were seeded into 24-well plates at the density of 1×10^4^ cells/well. After 24 h, the cells which had reached 80% confluence were exposed to 200 μg/ml of coumarin-6-loaded PEG-PLA micelles or coumarin-6-loaded mixed micelles and incubated for 15 min/1 h. The cells were then washed three times in cold PBS buffer and visualized under fluorescent microscope (Leica DMI 4000B, Leica, Germany).

For quantitative analysis, A549 cells were seeded into 6-well plates at the density of 1×10^5^ cells/well. After incubation with coumarin-6-loaded micelles, as described above, the cells were trypsinized and centrifuged at 2000 rpm for 4 min to obtain a cell pellet, which was subsequently resuspended in PBS and analyzed with a flow cytometer (FACSCalibur, BD Biosciences, NJ, U.S.).

### 2.7 Sub-cellular localization of the mixed micelle

Triple-labeling was made to determine which sub-cellular organelles were involved in the cytoplasmic distribution of the micelle. The localization of coumarin-6-labeled micelles was visualized with fluorescence markers specific to organelles, including LysoTracker Red and Hoechst 33342. A549 cells were seeded into a glass-bottom culture dish and cultured for 24 h and then incubated with 200 μg/ml of coumarin-6-loaded PEG-PLA micelles and coumarin-6-loaded mixed micelles. Then the cells were further treated with 50 nM LysoTracker Red (30 min) and 10 μg/ml of Hoechst 33342 (10 min) to visualize the lysosomes and nuclei, respectively. Finally, the cells were washed three times with cold PBS and observed under a laser scanning confocal microscope (Leica TCS SP5).

### 2.8 Inhibitory effect on tumor cells

To measure the cytotoxicity of the two PTX formulations and evaluate the role of the VE-TPGS in reversing MDR, A549 cells, KB cells, and KBv cells were seeded at a density of 5×10^3^ cells/well into 96-well plates. After 24 h incubation, the media were substituted with different PTX formulations at a series of concentrations (0.001 to 10 μg/ml) for 72 h, including the PTX solution (1.0% DMSO in PBS), PTX-loaded PEG-PLA micelles, and PTX-loaded mixed micelles. Cell viability was measured with an MTT assay and the absorbance of each well was determined at 570 nm with a microplate reader (BioTek Synergy TM2; Winooski, VT, U.S.). To evaluate the effect of the excipients, the cytotoxicity of the blank mixed micelle was also tested with the method described above at concentrations ranging from 0.1 to 1000 μg/ml. The results are here expressed as percent cell viabilities relative to that of cells incubated in the culture medium without drugs or excipients.

### 2.9 Tumor spheroids experiments

#### 2.9.1 Tumor spheroid model

An A549 tumor spheroid model was built with the lipid overlay method as reported previously [31,32]. Briefly, a 96-well plate was initially coated with 2% (w/v) agarose gel to prevent cell adhesion. Then A549 cells were seeded at a density of 1×10^4^ cells/well, gently shaken for 5 min, and incubated at 37°C for 7 days. Uniform and compact multicellular spheroids were chosen for subsequent studies.

#### 2.9.2 Penetration in tumor spheroid

To compare the tumor-penetrating ability of the PEG-PLA micelle and the mixed micelle on solid tumors *in vitro* and to confirm the effect of the mixed micelles on overcoming MDR, the tumor spheroids were incubated with the coumarin-6-loaded PEG-PLA micelles and the coumarin-6-loaded mixed micelles, diluted by a DMEM medium to the micelle concentration of 200 μg/ml at 37°C for 4 h. Then, the tumor spheroids were rinsed with cold PBS and fixed with 4% paraformaldehyde before subjection to a laser scanning confocal microscopy analysis (LSM510, Leica).

### 2.10 Statistical analysis

The results are here given as mean ± standard deviation. The statistical difference between the two mean values was calculated using a two-tailed Student’s t-test. The values were considered statistically significant if *P* < 0.05.

## Results and Discussion

### 3.1 Amphiphilic block ratio of PEG-PLA

As reported previously, increasing the chain length of the hydrophobic block may result in a significant increase in the hydrophobic drug-loading capacity of the copolymer [26]. Compared with those of group 1, increased EE% and DL% of group 2 at high feeding ratio (8:50 and 15:50) were as expected (Tables 2 and 3). However, a comparison of the results of groups 2 and 3 revealed an unexpected tendency: PEG_2000_-PLA may have a block ratio optimal for incorporating PTX, especially when maximum EE% and DL% are required. At a low feeding ratio (2:50), the PTX-loading capacity of the three groups did not differ significantly. As the feeding ratio increased, the EE% of groups 1 and 3 both dropped significantly, and the DL% of groups 1 and 3 were significantly lower than theoretical values. These results suggested that group 2 (M.W.3678, PEG_2000_/PLA = 54/46), in which the amphiphilic block ratio was close to 50/50, produced the best drug-loading capacity. The ratio was also similar to that observed in research on Genexol-PM [6]. Finally, an amphiphilic block ratio of 54/46 was chosen for preparation of the PEG-PLA copolymer as one of the materials to incorporate PTX.

**Table 2:** Block ratio and encapsulation efficiency (EE%).

Feeding ratio	EE%		
	M.W.2847	M.W.3678	M.W.5230
**2:50**	89.80	89.70	86.00
**8:50**	64.75	90.25	76.55
**15:50**	36.58	95.77	48.05

Values are presented as mean ± SD (n = 3).

**Table 3:** Block ratio and drug-loading coefficient (DL%).

Feeding ratio	DL%			
	Theoretical value	M.W.2847	M.W.3678	M.W.5230
**2:50**	3.80	3.47	3.46	3.33
**8:50**	13.80	9.39	12.62	10.91
**15:50**	23.10	9.89	20.32	12.60

Values are presented as mean ± SD (n = 3).

### 3.2 Formulation

**Fig 1:**
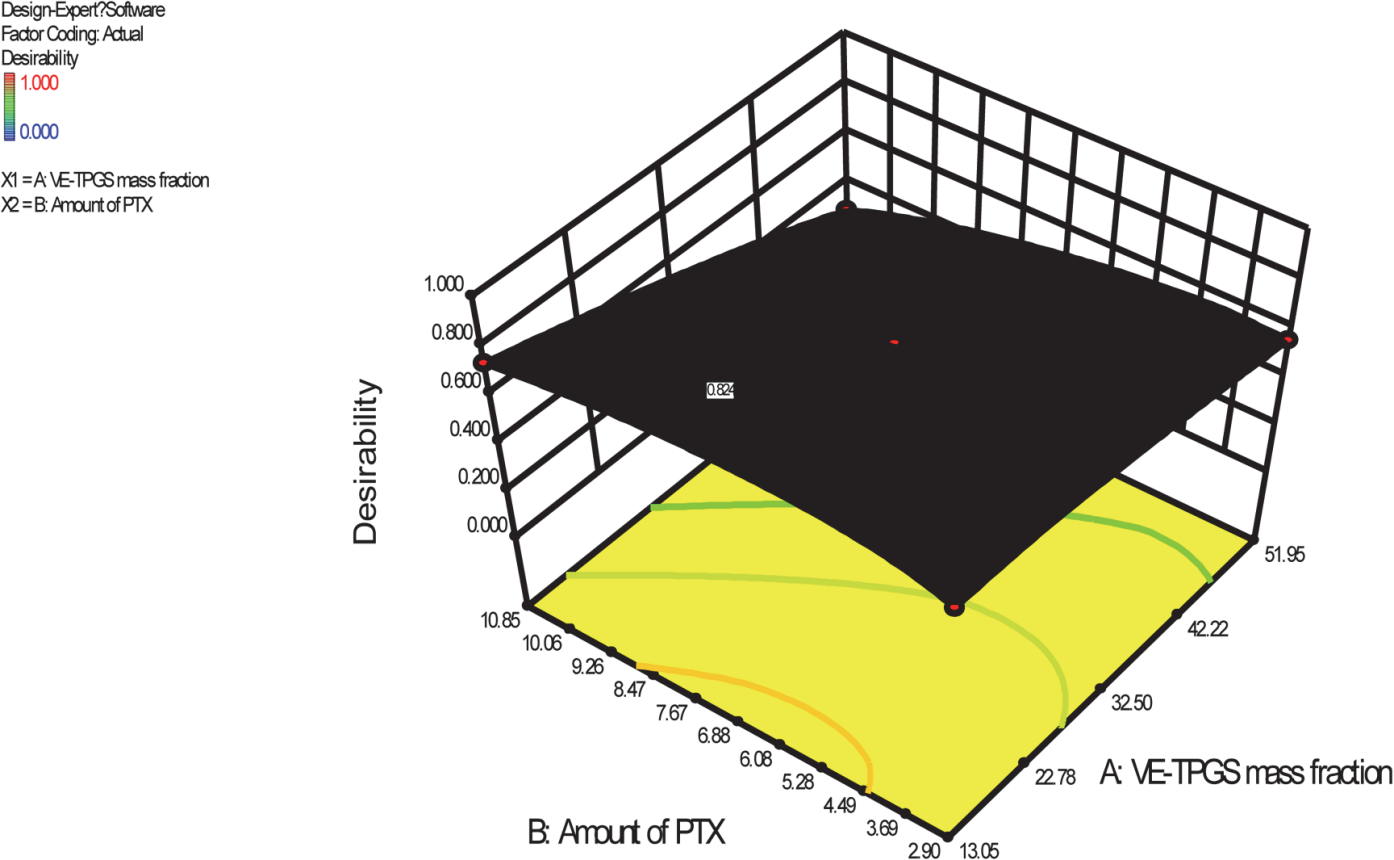
Response 3D surface in terms of desirability as suggested by the simulation for the optimization work.

The numerical optimization module in Design-Expert was used to search for a combination of factor levels that satisfied the requirements placed on each of the responses and factors. For better drug-loading capacity and micelle stability, the requirements were set to maximize the EE% and DL%, and minimize the particle size and PTX Pre%. Through a desirability function, the module predicted the ranges of variables where the optimal formulation may occur (Fig. 1 shows the response 3D surface, based on the CCD model). The optimal factors and predicted responses were verified by experiments (Table 4). The optimal VE-TPGS mass fraction was 13% (w/w), and the optimal amount of feeding PTX was 10 mg. The low bias showed that the CCD was a good tool for micelle formulation. The DL% of the mixed micelle for the PTX was determined as 24.32%, which was greater than that of Genexol-PM (16.67%). The results suggested that the mixed micelle had significantly better PTX-loading capacity than Genexol-PM. This may be caused by the enhancement of the lipophilicity of the inner core of the micelle. Tocopherol was regarded as a strong lipophilic substance [22].

**Table 4:** Observed and predicted values for the optimized formulation.

Factor	Optimized level	
A: VE-TPGS mass fraction (%)	13	
B: Amount of PTX (mg)	10	
C: Hydration temperature (℃)	25	
D: Amount of water (ml)	10	
E: Organic solvent	Acetonitrile	
F: Aqueous medium	Deionized water	
Response	Predicted	Observed
Y_1_: size (nm)	14.48	16.36
Y_2_: DL (%)	25.44	24.32
Y3: EE (%)	92.69	94.04
Y_4_: PTX precipitated (%)	20.46	18.02

### 3.3 Characterization of PTX-loaded mixed micelle

#### 3.3.1 Particle size, zeta potential, and morphology

**Fig 2:**
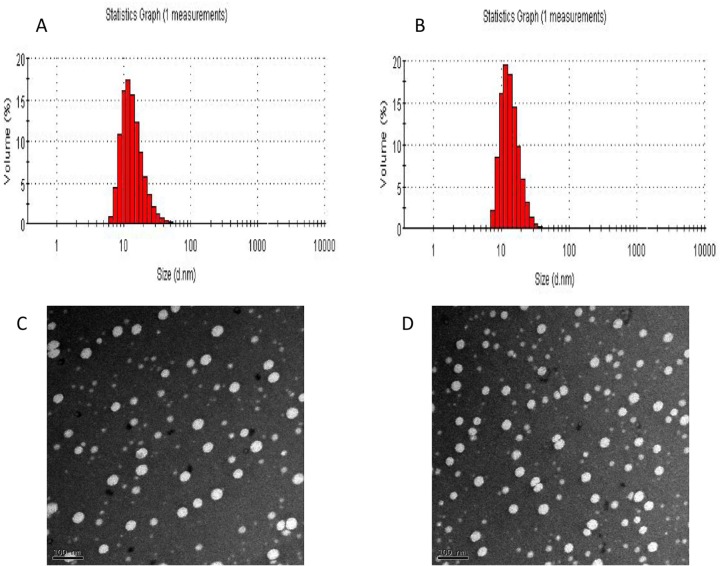
Micelle size and size distribution of (A) PEG-PLA-PTX micelles and (B) PV-PTX (PEG-PLA/Vitamin E-TPGS-PTX) mixed micelles. Transmission electron microscope (TEM) images of (C) PEG-PLA-PTX micelles and (D) PV-PTX mixed micelles (100,000×).

The mean diameters and zeta potentials of the PEG-PLA micelles and mixed micelles were 22.46±0.54 nm, -4.36 mv and 16.36±0.78 nm, -2.79 mv, respectively. Fig. 2A and 2B show the size distributions. The addition of VE-TPGS caused there to be smaller mixed micelle particles than PEG-PLA micelles but had no visible effect on micelle morphology. The PEG-PLA micelle and the mixed micelle both exhibited spherical shapes with homogeneous particle sizes under TEM (Fig. 2C and 2D). This was consistent with the results measured by laser scattering technique. The small particle size (<200 nm) could help to circumvent the RES uptake and facilitate extravasations from leaky capillaries [33].

#### 3.3.2 Critical micelle concentration determination

**Fig 3:**
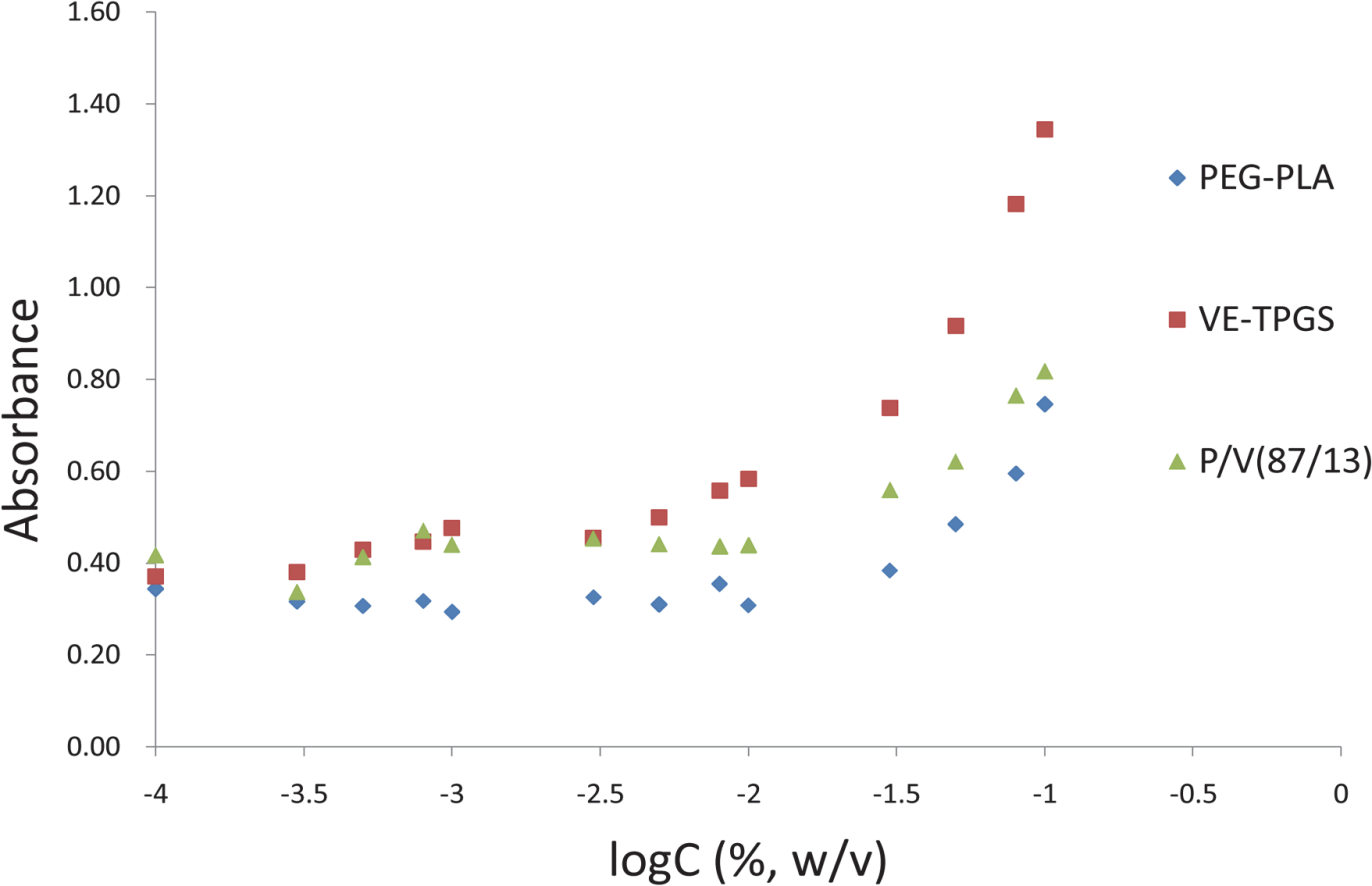
UV intensity plot of I_2_ versus logarithm of the polymer mass concentration (n = 3).

Iodine was used as a hydrophobic probe to monitor the formation of the mixed micelles. Because it is a small hydrophobic molecule, iodine was incorporated into the hydrophobic cores of the micelles. This caused a conversion of I_3_^−^ to I_2_, which maintained the saturated concentration of I_2_ solution [29]. The CMC value of each group was determined by plotting the absorption intensity against the logarithm of the polymer mass concentration (Fig. 3). The CMC value of the mixture (PEG-PLA/VE-TPGS = 87/13, w/w) was calculated as 0.0079% (w/v), a value between that of PEG-PLA (0.0107%, w/v) and VE-TPGS (0.0032%, w/v). The results indicated that the two copolymers had formed a mixed micelle instead of existing independently. Due to its low CMC, the mixed micelle had a greater anti-dilution capacity and stability than the PEG-PLA micelle [34].

#### 3.3.3 Physical status of PTX in the mixed micelle

**Fig 4:**
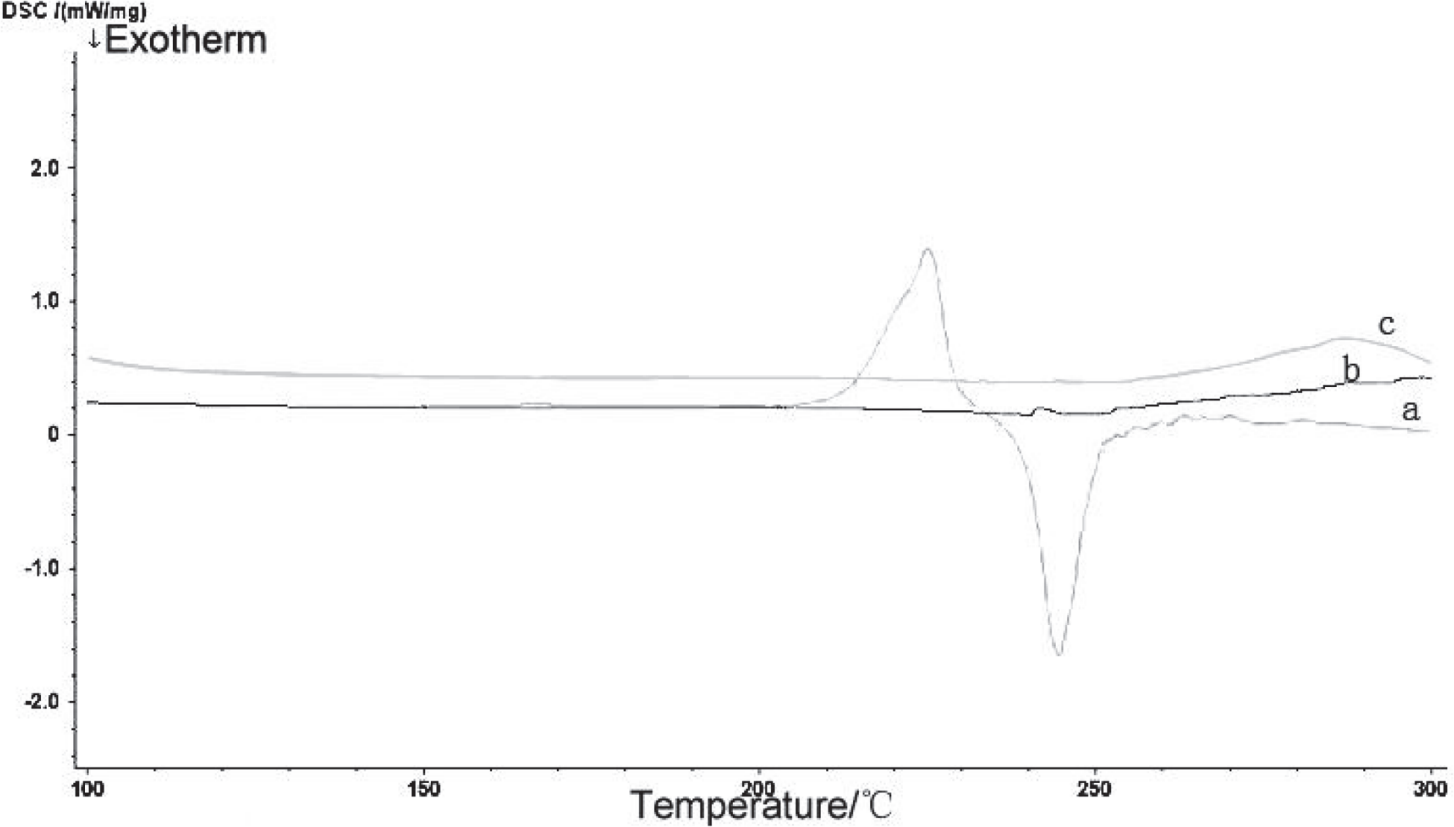
Differential scanning calorimetry (DSC) thermograms of (a) PTX, (b) blank mixed micelles, and (c) PTX-loaded mixed micelles.

The encapsulation of PTX into the core of the mixed micelle was confirmed by DSC. Fig. 4 shows the thermo grams of (a) PTX, (b) blank mixed micelle, and (c) PTX-loaded mixed micelle. Note the endothermic melting peak of pristine PTX at 225°C. The peak disappeared in the thermo gram of the PTX-loaded mixed micelle, suggesting that the PTX incorporated in mixed micelle existed as an amorphous molecular dispersion or a solid solution in the polymer matrix.

#### 3.3.4 Release behavior of micelle ***in vitro***

**Fig 5:**
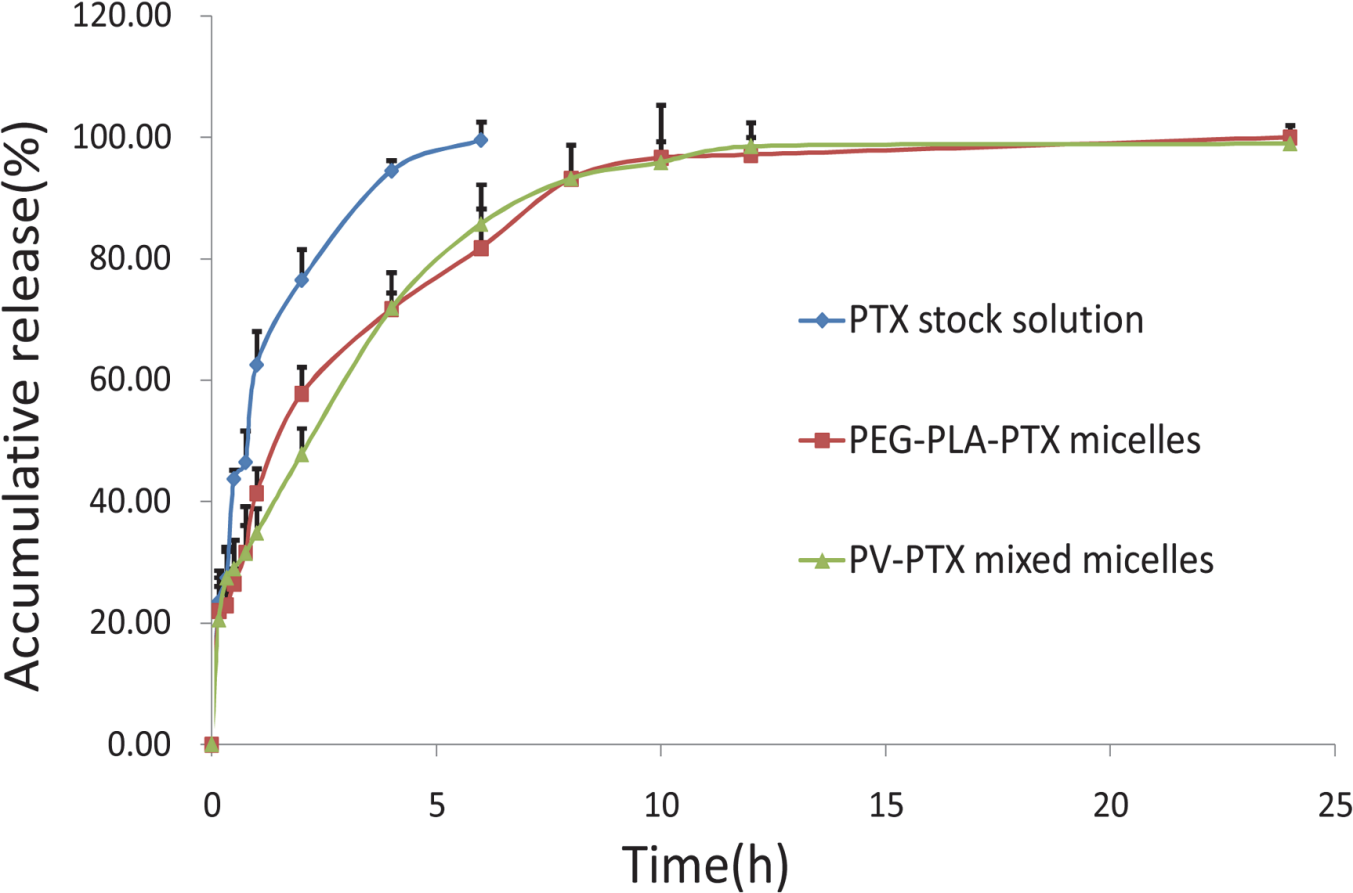
Release profiles of PTX from PTX formulations in a 0.5% Tween 80 medium at 37°C (n = 3).

As shown in Fig. 5, more than 80% of PTX in the stock solution was released within 2 h, which suggested that PTX could freely diffuse through the dialysis membrane. The *in vitro* release profile of the mixed micelle was similar to that of the PEG-PLA micelle, suggesting that the addition of VE-TPGS as part of the vehicle did not influence the *in vitro* release behavior of the PTX-loaded PEG-PLA micelle. The release behavior of the PTX from the mixed micelle exhibited a biphasic pattern with a characteristic fast initial release during the first 3 h, followed by a slower, continuous release. Accordingly, the *in vitro* release profile of the PTX from the polymeric micelle is largely affected by the hydrophobic properties of its inner core [35]. The strong hydrophobic interaction between the PTX and the tocopherol segment of the VE-TPGS may have resulted in longer diffusion time of the PTX, which finally led to a lower release rate than the PEG-PLA micelle during the first 4 h.

### 3.4 Cellular association of coumarin-6-labeled mixed micelle

**Fig 6:**
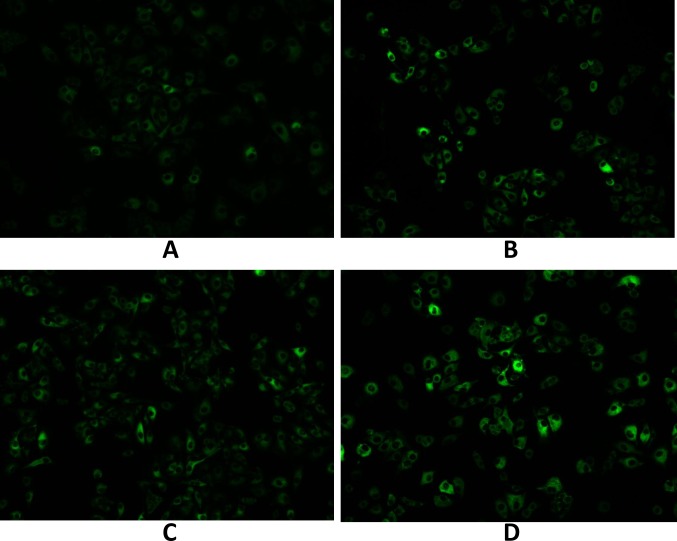
A549 cell uptake (A) after 15 min and (B) after 1 h of culture with coumarin-6-loaded (a fluorescence probe, green) PEG-PLA micelles and (C) after 15 min and (D) after 1 h of culture with coumarin-6-loaded mixed micelles.

**Fig 7:**
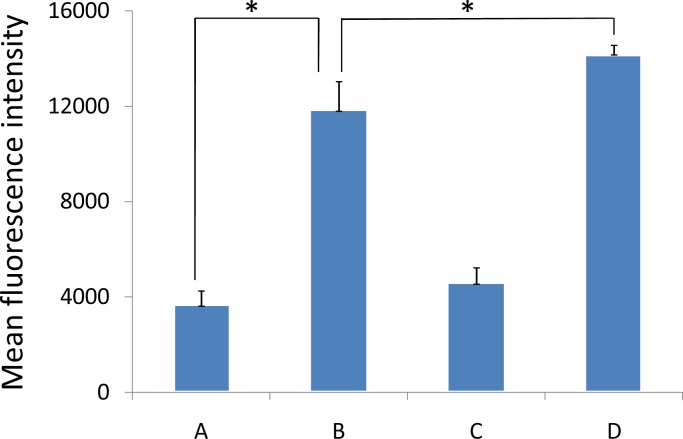
Quantitation of mean fluorescence intensity of coumarin 6 in A549 cells (A) after 15 min and (B) after 1 h of culture with coumarin-6-loaded PEG-PLA micelles and (C) after 15 min and (D) after 1 h of culture with coumarin-6-loaded mixed micelles. Data are presented as mean±SD (n = 3). *P<0.05.

Coumarin-6 was used as a fluorescent probe to study the cell uptake characteristics of the mixed micelle. Qualitative fluorescent images (Fig. 6) showed that the cellular association of the coumarin-6-labeled micelles in A549 cells displayed a time dependent behavior. At the same micelle concentration, the fluorescence intensity of the A549 cellular associated mixed micelles was significantly higher than that of the PEG-PLA micelles after incubation for 15 min and after incubation for 1 h (200 μg/ml, *P*<0.05) (Fig. 7). The results suggested that the mixed copolymers could facilitate the uptake of micelles by A549 cells and that VE-TPGS may play a role in this facilitation. Improving the fluidity of the cell membrane as reported previously [36] and overcoming MDR, which function of VE-TPGS may have played a major role in the results. This requires confirmation.

### 3.5 Sub-cellular localization of the mixed micelle

**Fig 8:**
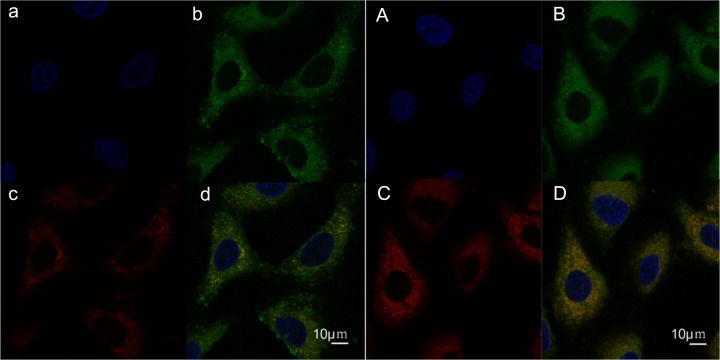
Confocal microscopy images showing the intracellular localization of coumarin-6-loaded PEG-PLA micelles in A549 cells. (a) nucleus, (b) PEG-PLA micelles, and (c) lysosomes were distinguished using Hoechst 33342 (blue), coumarin 6 (green), and Lyso Tracker Red (red). (d) Yellow represents the colocalization of Lyso Tracker Red with Green (coumarin-6-loaded PEG-PLA micelles). A, B, C, and D show the localization of coumarin-6-loaded mixed micelles.

Fig. 8 shows the sub-cellular localization of the coumarin-6-labeled PEG-PLA micelles and the mixed micelles. The lysosomes were visualized as red fluorescence after staining A549 cells with LysoTracker Red, and the nuclei were visualized with blue fluorescence after Hoechst 33342 staining. The coumarin-6-labeled micelles are shown as green fluorescence. The colocalization of micelles with the organelle-selective fluorescence markers are shown in yellow. After incubation for 30 min, the two micelles were both found in the cytoplasm and mainly localized in the lysosomes rather than nucleus. This indicated that either the PEG-PLA micelles or the mixed micelles had been delivered to the lysosomes. The similarity suggested that the VE-TPGS did not change the intracellular behavior of the PEG-PLA micelle.

### 3.6 Inhibitory effect on tumor cells

**Fig 9:**
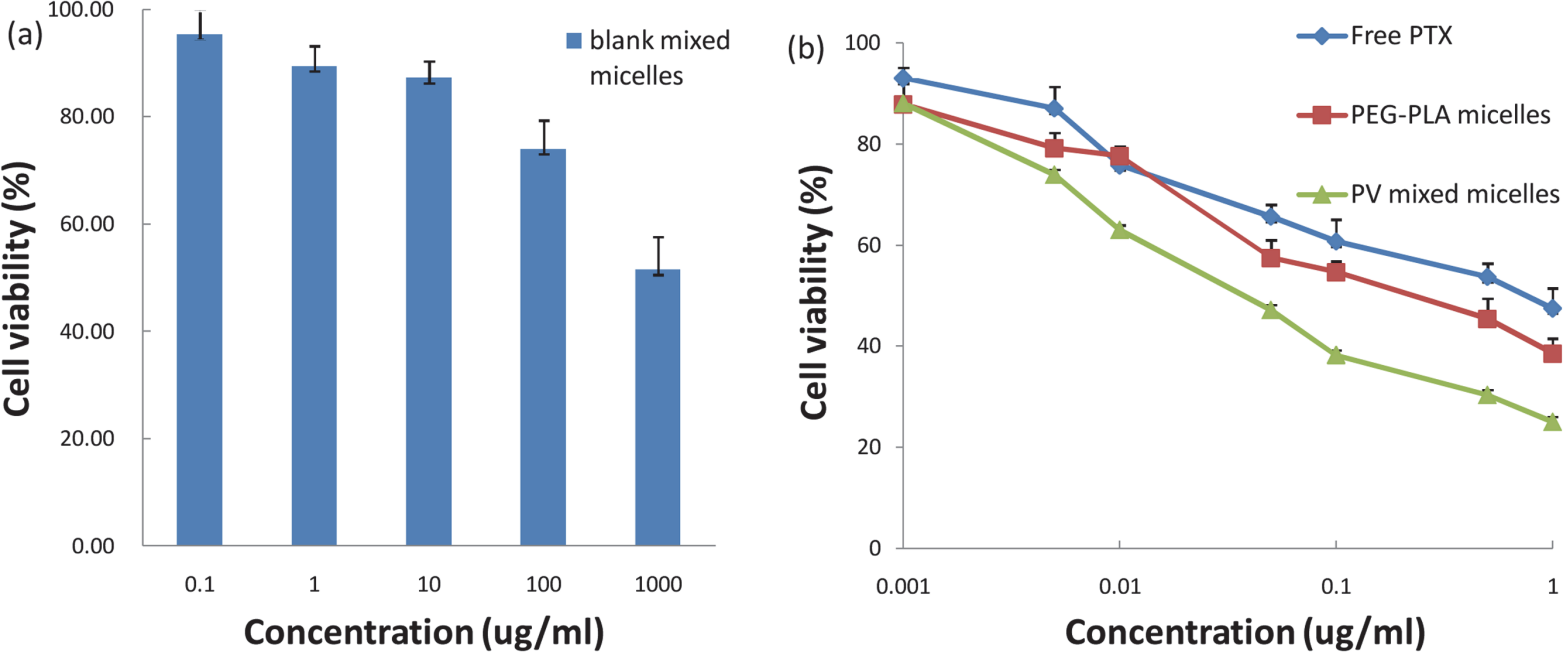
(a) Viability of A549 MDR tumor cells as a function of varying concentrations of blank mixed micelles; (b) *in vitro* cytotoxicity of various formulations of PTX against A549 cells (n = 6).

The biocompatibility of the materials *in vitro* was evaluated on A549 cells. As shown in Fig. 9A, the cytotoxicity of the blank mixed micelle was negligible at concentrations ranging from 0.1 to 100 μg/ml. When the concentration reached 1000 μg/ml, the cytotoxicity increased significantly. However, the value of the IC_50_ was still greater than 1 mg/ml, which meant that the PEG-PLA and the VE-TPGS were both biocompatible and that the mixed micelle could be used safely to deliver PTX.

The cytotoxic effect of the PTX-loaded micelle was determined employing the A549 cell line, at the beginning (Fig. 9B). A549 cells are MDR tumor cells which overexpress P-gp on their cell membrane [37,38]. The groups were sorted by IC_50_ values as follows: mixed micelle < PEG-PLA micelle < Free PTX (Table 5). There was a significant difference between the mixed micelle group and the PEG-PLA micelle group (*P*<0.01). This showed that the mixed micelle group had a significantly greater cytotoxic effect on A549 cells.

**Table 5:** IC_50_ of PTX formulations against different tumor cell lines.

Formulation	IC_50_ (μg/ml)		
	A549	KB	KBv
**Blank mixed micelles**	> 1000		
**Free PTX**	0.55 ± 0.07	0.10 ± 0.02	25.00 ± 3.71
**PEG-PLA micelles**	0.22 ± 0.02	0.05 ± 0.01	7.08 ± 1.28
**PV mixed micelles**	0.05 ± 0.02	0.03 ± 0.01	0.82 ± 0.09

Values are presented as mean ± SD (n = 6).

**Fig 10:**
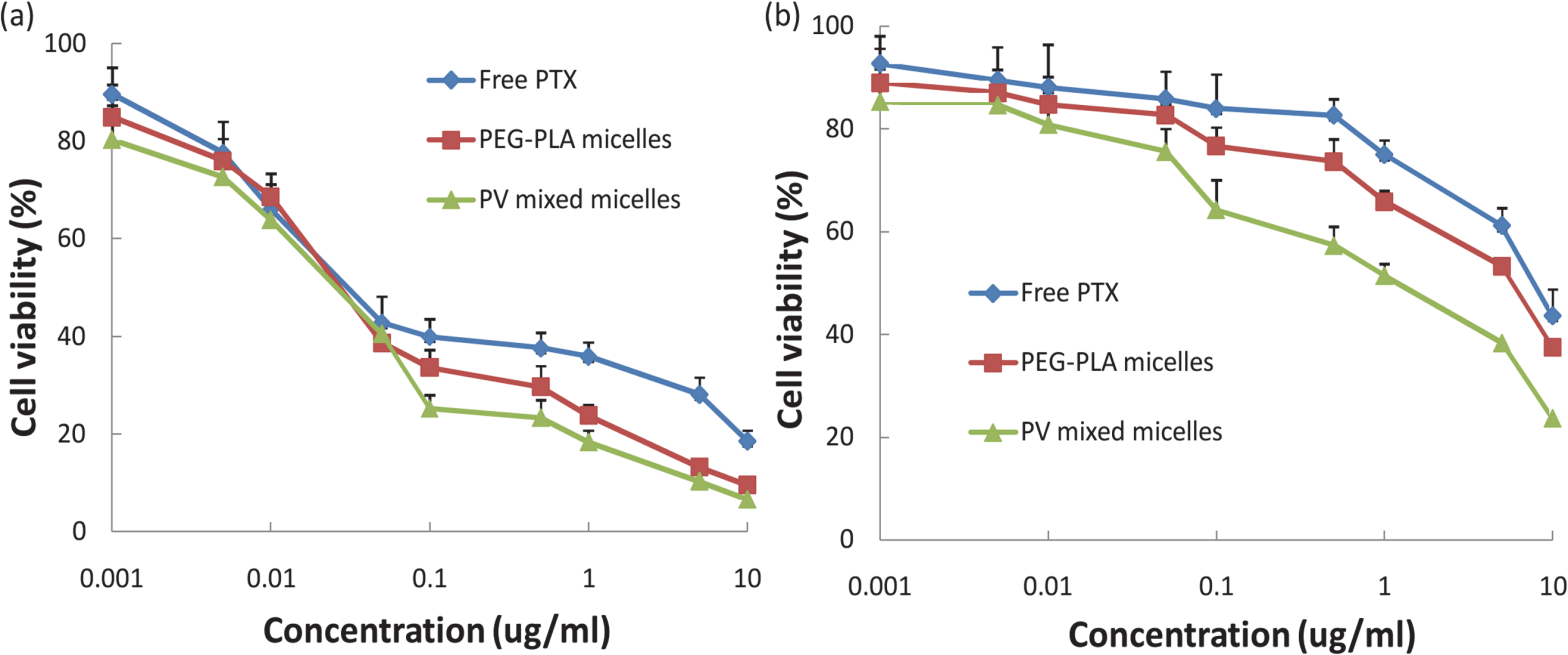
*In vitro* cytotoxicity of various formulations of PTX against (a) KB tumor cells and (b) KBv tumor cells (n = 6).

To confirm the effect of the VE-TPGS on the result by its function in reversing the MDR of A549 tumor cells, MTT assays of the human oral epithelial cancer cell line KB and its vinblastine-resistant subclone KBv were designed. KB cells, which were sensitive to PTX, were used as control, to compare with the MTT assay results of the KBv cells. After 72 h of incubation, the PEG-PLA micelles and the mixed micelles both exhibited a strong cytotoxicity against the proliferation of KB cells (Fig. 10A). The IC_50_ values were 0.05 μg/ml for the PEG-PLA micelles and 0.03 μg/ml for the mixed micelles (Table 5). However, as shown in Fig. 10B, at various concentrations, the mixed micelles all exhibited a stronger inhibitory effect on the growth of KBv cells than the PEG-PLA micelles did. The IC_50_ values were 7.08 μg/ml for the PEG-PLA micelles and 0.82 μg/ml for the mixed micelles (Table 5). Unlike the sensitive KB cells, the MDR KBv cells showed a considerably superior response to the cytotoxicity of the mixed micelles than to that of the PEG-PLA micelles (*P<0*.*01*); this observation was consistent with the results regarding the A549 cell line. These results demonstrated that the addition of VE-TPGS could help the PEG-PLA micelle enhance the cytotoxicity against MDR cancer cells. The overexpressed P-gp in A549 and KBv cells led to increased drug efflux, and PTX has been confirmed to be a P-gp substrate [13,14]. As reported previously, VE-TPGS inhibits the efflux of PTX through allosteric regulation of the P-gp ATP enzyme, which blocks the energy source of the P-gp [25]. This might explain the hypersensitive effect of VE-TPGS on A549 and KBv cells.

### 3.7 Penetration of tumor spheroid

**Fig 11:**
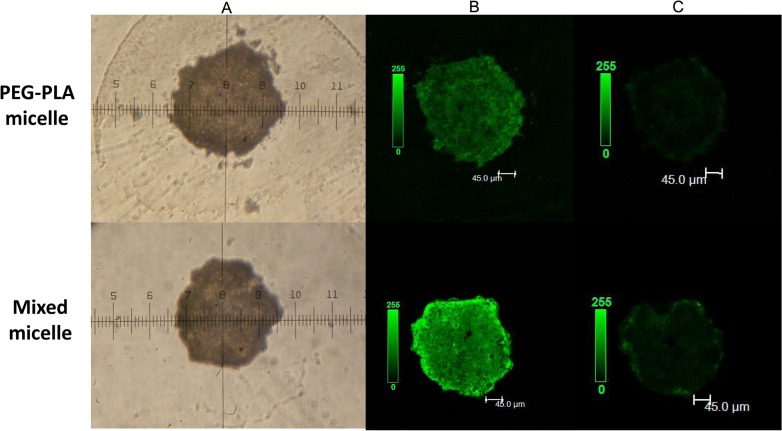
(A) A549 tumor spheroid at 7 days after cell seeding. Images were acquired at 10×. (B) Z-stack confocal microscope images of A549 tumor spheroids incubated with coumarin-6-loaded different micelles. (C) Images were obtained at 100 μm along the Z-axis from the top of the spheroid.

Three-dimensional multicellular tumor spheroids of the A549 cells were established to mimic the solid tumors *in vivo* and evaluate the penetrating ability of the micelles on solid tumors. As shown in Fig. 11 (confocal microscopy analysis), mixed micelles were distributed more extensively than the PEG-PLA micelles in the A549 tumor spheroids in Z-stack confocal microscope images (B). The images obtained at 100 μm along the Z-axis from the top of the spheroid (C) showed that the mixed micelles penetrated even deeper than the PEG-PLA micelles, which had reached the inner core of the tumor spheroid. These results confirmed the conclusion that the mixed micelle could improve the antitumor efficacy of the PTX by reversing the MDR.

## Conclusion

In this study, we developed a new mixed micelle to improve the antitumor efficacy of Genexol-PM. The new micelle consists of PEG-PLA (the original vehicle of Genexol-PM) and VE-TPGS. This micelle is a suitable vehicle for the poorly soluble anticancer drug PTX. VE-TPGS was chosen for its ability to overcome the MDR of tumor cells. The amphiphilic block ratio of PEG-PLA was found to be critical to the mixed micelle formation; we think that the ratio PEG_2000_/PLA = 54/46 is the best option. A CCD was used to optimize the formulation: The result was a mixed micelle with a particle size of around 16.36 nm. Surprisingly, compared with Genexol-PM, the mixed micelle improved the PTX-loading capacity significantly (DL% = 24.32%) and had a lower CMC, i.e., a stronger anti-dilution capacity. For MDR tumor cells (A549, KBv), PTX-loaded mixed micelle displayed noticeable antitumor efficacy. It enhanced the cell uptake significantly by overcoming MDR. These results showed that the PEG-PLA/Vitamin E-TPGS mixed micelle may be better than Genexol-PM as a vehicle of PTX in the treatment of MDR tumors.

## Supporting Information

S1 File NC3Rs ARRIVE Guidelines Checklist. (PDF)
