# Correction: Adding Vitamin E-TPGS to the Formulation of Genexol-PM: Specially Mixed Micelles Improve Drug-Loading Ability and Cytotoxicity against Multidrug-Resistant Tumors Significantly

**DOI:** 10.1371/journal.pone.0127343

**Published:** 2015-05-07

**Authors:** The PLOS ONE Staff

In the PDF, Figs 6 and 7 incorrectly appear as duplicates of Figs 10 and 11. The HTML version and figure legends are correct. The publisher apologizes for the error. Please see the correct versions of Figs 6 and 7 here.

**Fig 6:**
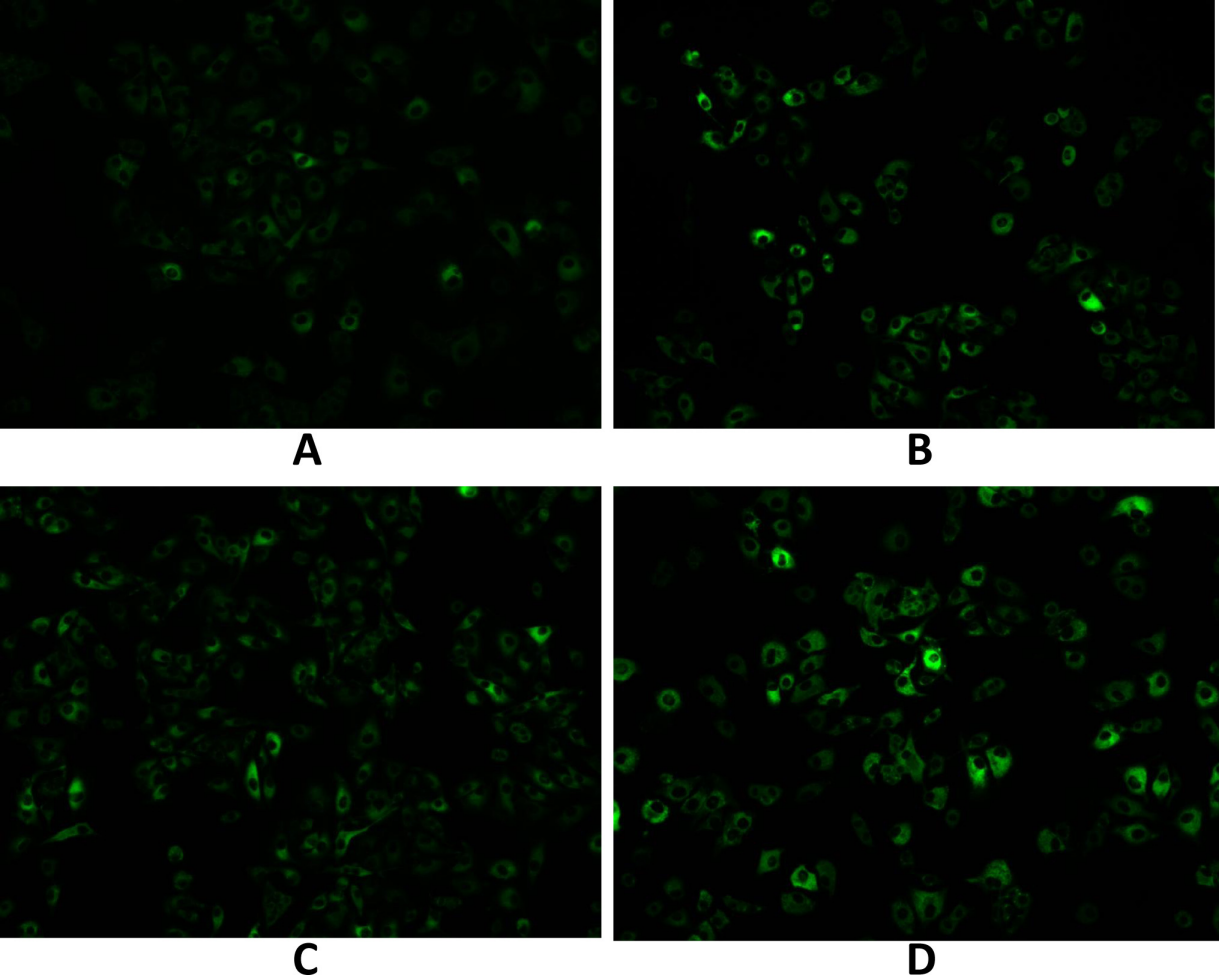
A549 cell uptake (A) after 15 min and (B) after 1 h of culture with coumarin-6-loaded (a fluorescence probe, green) PEG-PLA micelles and (C) after 15 min and (D) after 1 h of culture with coumarin-6-loaded mixed micelles.

**Fig 7:**
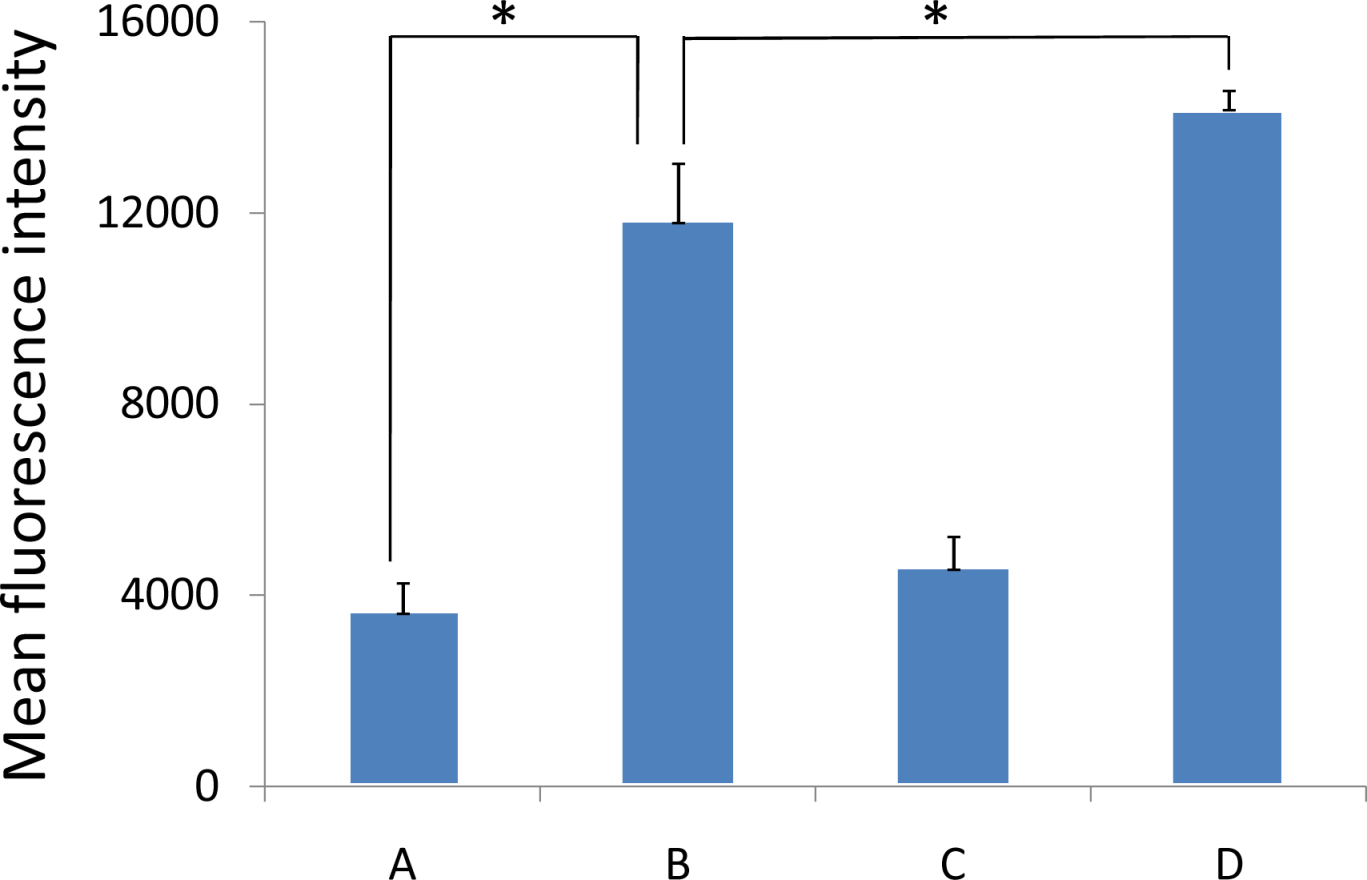
Quantitation of mean fluorescence intensity of coumarin 6 in A549 cells (A) after 15 min and (B) after 1 h of culture with coumarin-6-loaded PEG-PLA micelles and (C) after 15 min and (D) after 1 h of culture with coumarin-6-loaded mixed micelles.
